# Association between serum low-density lipoprotein cholesterol and metabolic syndrome in a working population

**DOI:** 10.1186/s12944-021-01500-1

**Published:** 2021-07-18

**Authors:** Saibin Wang

**Affiliations:** Department of Respiratory Medicine, Affiliated Jinhua Hospital, Zhejiang University School of Medicine, Jinhua Municipal Central Hospital, No. 365, East Renmin Road, Jinhua, 321000 Zhejiang Province China

**Keywords:** LDL-C, Metabolic syndrome, Dyslipidemia, Lipid

## Abstract

**Background:**

The studies, investigating the association of low-density lipoprotein cholesterol (LDL-C) with metabolic syndrome (MetS) are limited with controversial conclusions. Therefore, this study aimed at revealing the specific relationship between the serum LDL-C levels and MetS prevalence in a large working population.

**Methods:**

Secondary data analysis of a cross-sectional study, conducted between 2012 and 2016 in Spain on participants aged within the range of 20–70 years, involved 60,799 workers. Logistic regression analysis was applied to evaluate the association between the levels of serum LDL-C and MetS prevalence.

**Results:**

Among the 60,799 workers, the prevalence of MetS was 9.0%. The odds ratios (95% confidence intervals) of MetS prevalence were 1.27 (1.16–1.39) and 1.53 (1.41–1.65) for the individuals with the LDL-C levels in lower (< 103.8 mg/dL) and upper (> 135.8 mg/dL) tertiles as compared to those with the LDL-C levels in middle tertile (103.8–135.8 mg/dL) in the studied population. Similarly, a U-shaped relationship was also observed in male cohort. The serum LDL-C levels associated with the lowest risk of current MetS were 113.6 mg/dL and 117.6 mg/dL in the overall studied population and male cohort, respectively. The female workers with the levels of LDL-C higher than 135.0 mg/dL had an increased prevalence of MetS (*P* < 0.05).

**Conclusions:**

The low and high levels of serum LDL-C were associated with an increased prevalence of MetS in the working population and in male workers. Only the high (> 135.0 mg/dL) levels of LDL-C increased MetS prevalence in female workers.

## Background

Metabolic syndrome (MetS) consists of a cluster of metabolic abnormalities, including abdominal obesity, insulin resistance, systemic hypertension, atherogenic dyslipidemia, and hyperglycemia [1–4]. It has a high prevalence worldwide and can also increase the risk of many other disorders, such as cardiovascular disease (CVD), cancer, liver disease, type II diabetes mellitus, and mental illness [1, 2, 5–8].

Dyslipidemia is a characteristic feature of MetS, and is used as a biomarker for its diagnosis [1]. The high levels of triglycerides and low levels of high-density lipoprotein cholesterol (HDL-C) are also used to diagnose MetS according to the criteria of National Cholesterol Education Program-Adult Treatment Panel III (NCEP-ATP III) [9]. Apart from the association of triglycerides and HDL-C with MetS, several studies also tried to reveal the relationship between other blood lipid components and MetS. For example, the serum non-HDL-C levels (> 118 mg/dL or < 247 mg/dL) are related to the rising incidence of MetS [9]. Despite the fact that the low-density lipoprotein cholesterol (LDL-C) has been a well-known indicator for the pathogenesis of CVD [10], Hajian-Tilaki et al. reported that the levels of serum LDL-C were not significantly related to MetS prevalence based on a cross-sectional study, which was conducted in Iranian adults (*n* = 1000) aged 20–70 years [11]. However, a prospective study in China by Wang et al., conducted on a cohort of 4542 participants aged within the range of 19–80 years, suggested that the rise in serum LDL-C levels increased the risk of MetS [12]. Given the contrasting results in the above studies regarding the relationship between serum LDL-C levels and MetS, there is still the need of discovering a specific relationship between them in a larger population.

Therefore, in order to explore the specific association between the serum LDL-C levels and prevalence of MetS, this study was conducted as a secondary analysis using a large sample of a working population.

## Methods

### Study population and data collection

This study was conducted as a secondary analysis of data from a cross-sectional study [13]. The used data in this analysis were obtained from a public database (www.Datadryad.org). Romero-Saldaña et al. transferred the ownership of the original data to the Datadryad website, and this website (database) permits users to freely download raw data for secondary analysis (Dryad data package used in this study: Romero-Saldaña, Manuel et al. (2018), Data from: Validation of a non-invasive method for the early detection of metabolic syndrome: a diagnostic accuracy test in a working population, Dryad, Dataset, 10.5061/dryad.cb51t54). A total of 60,799 participants (aged within the range of 20–70 years, 57.3% male), belonging to different socioeconomic sectors (e.g., healthcare services and public administration) were voluntarily recruited from Balearic Islands, Spain during the periodic assessment of their health, using random sampling between 2012 and 2016 [13]. In the study, 12.6% workers were excluded for their refusal to participate [13]. As stated by Romero-Saldaña et al. in the previously published article, the study was conducted with the Declaration of Helsinki principles, and written informed consents were obtained from participants [13].

The variables investigated in this study included baseline characteristics, anthropometric measurements, and laboratory tests. All the anthropometric parameters were measured in triplicates by well-trained staff, and then their average was recorded. A 12-h overnight fasting venous blood sample was used for laboratory tests. The laboratory tests followed the standard procedures for clinical biochemical laboratories, as described previously [13]. The following variables were collected for analysis: age (year), gender, smoking status (yes or no), body mass index (BMI), waist-to-height ratio (WHtR), waist circumference (WC), percentage of body fat (BF), a body shape index (ABSI), systolic blood pressure (SBP), and diastolic blood pressure (DBP). The laboratory variables included LDL-C, triglycerides, HDL-C, total cholesterol (TC), and fasting blood glucose (FBG) [13]. The NCEP-ATP III definition was used to diagnose MetS in this study. The participants were diagnosed with MetS when they met at least three of the following: (i) WC more than 102 cm in men or more than 88 cm in women; (ii) triglycerides more than 150 mg/dL; (iii) HDL-C less than 40 mg/dL in men or less than 50 mg/dL in women; (iv) BP more than 130/85 mmHg; (v) FBG more than 100 mg/dL [1].

### Statistical analysis

The data was expressed as number (percentage) or mean ± standard deviation according to characteristic variables (categorical or continuous variables). Unpaired *t*-test or Pearson’s chi-squared test was used for the comparison between the two groups (MetS group and non-MetS group). In the present study, the serum LDL-C tertiles were used for the analysis based on a U-shaped relationship, which was observed between the levels of LDL-C and MetS prevalence. Multicollinearity test was performed. The following criteria were used for the selection of covariables for adjustment: introduction covariables in the basic model or removal of covariables from the full model led to a change in the regression coefficient (β) of LDL-C > 10%; the regression coefficient of covariables on dependent variable (current MetS) yielded a *P*-value < 0.1; or the covariables needed to be adjusted by clinical observations [9]. A multivariate analysis using a logistic regression model was performed for the assessment of association between serum LDL-C levels and MetS prevalence after the adjustment for potential confounders [14]. The statistical analysis and graphical representations were carried out using R version 3.5.1. A two-sided *P-*value of < 0.05 was considered statistically significant.

## Results

In this study, the prevalence of MetS was 9.0% (5587/60799, 95% confidence interval [CI], 8.9–9.4%) in the studied population. Among 34,827 male and 25,972 female participants, the prevalence of MetS was 11.8% (4097/34827) and 5.4% (1390/25872), respectively, based on the diagnostic criteria of MetS suggested by the NCEP-ATP III. Both the male and female participants, who developed MetS, were older than those without MetS (Table 1, *P* < 0.05). In addition, both the male and female participants with MetS had higher levels of serum LDL-C than those without MetS (*P* < 0.05).

**Table 1:** Characteristics of study participants by gender and current MetS

Characteristics	Men (***n*** = 34,827)			Women (n = 25,972)		
	MetS (***n*** = 4097)	Non-MetS	***P***-value	MetS (***n*** = 1390)	Non-MetS	***P***-value
		(***n*** = 30,730)			(***n*** = 24,582)	
Age, (year)	46.7 ± 9.3	39.5 ± 10.3	< 0.001	48.0 ± 9.2	39.0 ± 10.0	< 0.001
Smoking, n (%)			< 0.001			0.011
No	2369 (57.8)	19,712 (64.1)		982 (70.6)	16,559 (67.4)	
Yes	1728 (42.2)	11,018 (35.9)		408 (29.4)	8023 (32.6)	
SBP (mmHg)	138.0 ± 16.5	123.7 ± 14.8	< 0.001	132.8 ± 17.6	113.6 ± 14.3	< 0.001
DBP (mmHg)	84.1 ± 10.9	74.9 ± 10.4	< 0.001	82.0 ± 10.9	69.8 ± 10.0	< 0.001
BF (%)	31.3 ± 5.7	24.5 ± 5.8	< 0.001	43.7 ± 7.1	33.2 ± 6.4	< 0.001
ABSI (%)	7.8 ± 0.7	7.5 ± 0.6	< 0.001	7.2 ± 0.9	7.0 ± 0.7	< 0.001
BMI (kg/m^2^)	30.7 ± 4.6	26.4 ± 3.9	< 0.001	31.4 ± 6.0	24.7 ± 4.6	< 0.001
WC (cm)	100.4 ± 11.4	87.2 ± 8.2	< 0.001	89.7 ± 13.5	74.6 ± 8.9	< 0.001
WHtR	0.6 ± 0.1	0.5 ± 0.1	< 0.001	0.6 ± 0.1	0.5 ± 0.1	< 0.001
TC (mg/dL)	222.4 ± 41.7	193.5 ± 37.0	< 0.001	222.0 ± 40.2	191.3 ± 35.5	< 0.001
HDL-C (mg/dL)	43.1 ± 8.4	51.5 ± 6.9	< 0.001	47.1 ± 8.0	55.4 ± 9.0	< 0.001
LDL-C (mg/dL)	130.6 ± 43.7	120.6 ± 36.2	< 0.001	141.3 ± 39.6	119.3 ± 36.4	< 0.001
Triglycerides (mg/dL)	257.6 ± 145.9	107.7 ± 58.7	< 0.001	170.0 ± 92.0	83.2 ± 36.7	< 0.001
FBG (mg/dL)	107.4 ± 35.4	88.4 ± 17.3	< 0.001	104.2 ± 29.1	84.1 ± 13.1	< 0.001

*MetS* metabolic syndrome, *SBP* systolic blood pressure, *DBP* diastolic blood pressure, *BF* body fat, *ABSI* a body shape index, *BMI* body mass index, *WC* waist circumference, *WHtR* waist-to-height ratio, *TC* total cholesterol, *HDL-C* high-density lipoprotein cholesterol, *LDL-C* low-density lipoprotein cholesterol, *FBG* fasting blood glucose

**Fig. 1:**
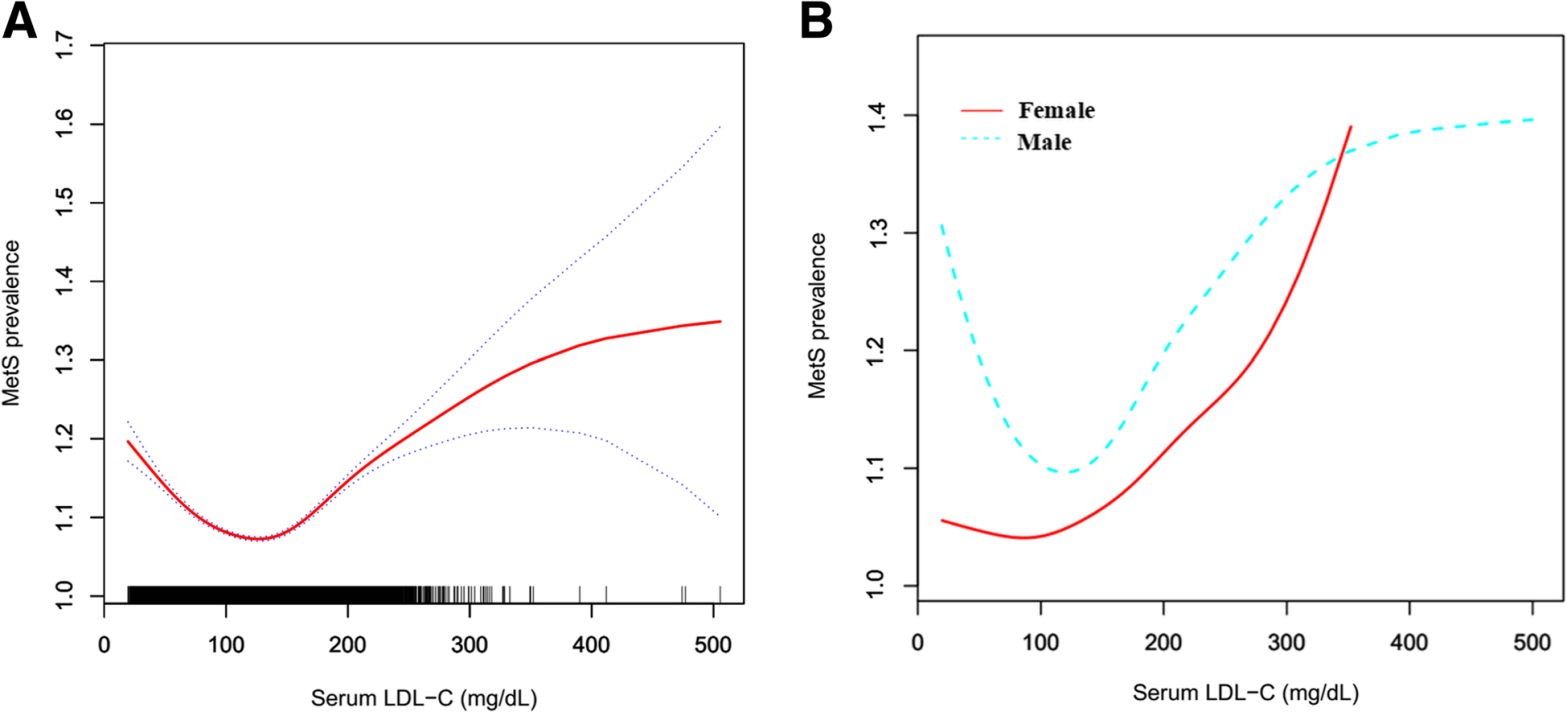
Smooth fitting curve shows a U-shaped association between the levels of serum LDL-C and MetS prevalence in the overall study population (**A**) and in male worker cohort (**B**, blue dotted line), with the inflection point is 113.6 mg/dL and 117.6 mg/dL, respectively. However, only increased levels of serum LDL-C (> 135.0 mg/dL) positively associated with MetS prevalence in female workers (**B**, solid red line). Adjusted for gender, age, BMI, DBP, ABSI, and smoking in (**A**), and adjusted for age, BMI, DBP, ABSI, and smoking in (**B**). LDL-C = low-density lipoprotein cholesterol; MetS = metabolic syndrome; BMI = body mass index; DBP = diastolic blood pressure; ABSI = a body shape index

In the multicollinearity test, four variables (age, BF, WHtR, and TC) were screened out. Except the age judged by clinical significance, the other three variables were excluded. In addition, the covariates of gender, BMI, DBP, ABSI, and smoking met the criteria needed to be adjusted in the multivariate regression analysis. A U-shaped relationship was observed between the serum LDL-C levels and MetS prevalence in the overall studied population (Fig. 1A). Furthermore, the tertile analysis confirmed a risky relationship of the lower or higher LDL-C levels with MetS prevalence in the multivariate regression analysis. After the adjustment for potential confounders based on different models (Model I adjusted for age; Model II adjusted for age, gender, BMI, DBP, ABSI, and smoking), the odds ratios (ORs) (95% CIs) of MetS were calculated to be 1.27 (1.16–1.39) in individuals with serum LDL-C levels < 103.8 mg/dL (lower tertile) and 1.53 (1.41–1.65) in those with serum LDL-C levels > 135.8 mg/dL (upper tertile) in Model II as compared to individuals with the LDL-C levels of 103.8–135.8 mg/dL (middle tertile) (Table 2). However, the relationship between the serum levels of LDL-C and MetS prevalence was influenced by gender, as revealed by the sensitivity analysis. Similarly, a U-shaped relationship between the two was observed in male workers [T1 vs. T2: 1.33 (1.19–1.48); T3 vs. T2: 1.50 (1.36–1.65)] (Fig. 1B, Table 2). Nevertheless, only the upper tertile of serum LDL-C levels (> 135.0 mg/dL) increased the prevalence of MetS in female workers [T1 vs. T2: 0.95 (0.78–1.15); T3 vs. T2: 1.52 (1.31–1.76)] (Table 2). Furthermore, the male population was grouped according to the threshold for the tertile of female population (T1: < 102.6 mg/dL, T2: 102.6–135 mg/dL, and T3: > 135.0 mg/dL). The results showed that, in addition to the increase in the prevalence of MetS by high levels of LDL-C (> 135 mg/dL), the low levels of LDL-C (< 102.6 mg/dL) also increased the prevalence of MetS (Table 2).

**Table 2:** Multivariable analysis of serum LDL-C levels and current MetS in study population, by tertile levels

LDL-C (mg/dL)	Odds Ratio (95% Confidence Interval) ***P***-value		
	Crude	Model I^a^	Model II^b^
**Subjects (*n*** **= 60,799)**			
T2 (103.8–135.8)	Ref.	Ref.	Ref.
T1 (< 103.8)	0.90 (0.83, 0.97) 0.005	1.22 (1.13, 1.32) < 0.001	1.27 (1.16, 1.39) < 0.001
T3 (> 135.8)	1.81 (1.70, 1.94) < 0.001	1.41 (1.32, 1.51) < 0.001	1.53 (1.41, 1.65) < 0.001
**Men (*n*** **= 34,827)**			
T2 (105.0–136.5)	Ref.	Ref.	Ref.
T1 (< 105.0)	1.02 (0.94, 1.12) 0.613	1.34 (1.22, 1.46) < 0.001	1.33 (1.19, 1.48) < 0.001
T3 (> 136.5)	1.73 (1.60, 1.88) < 0.001	1.44 (1.33, 1.57) < 0.001	1.50 (1.36, 1.65) < 0.001
102.6–135.0	Ref.	Ref.	Ref.
< 102.6	1.03 (0.94, 1.12) 0.518	1.35 (1.23, 1.48) < 0.001	1.34 (1.20, 1.49) < 0.001
> 135.0	1.71 (1.58, 1.85) < 0.001	1.41 (1.30, 1.53) < 0.001	1.46 (1.33, 1.61) < 0.001
**Women (n = 25,972)**			
T2 (102.6–135.0)	Ref.	Ref.	Ref.
T1 (< 102.6)	0.55 (0.47, 0.66) < 0.001	0.81 (0.68, 0.97) 0.018	0.95 (0.78, 1.15) 0.589
T3 (> 135.0)	2.06 (1.82, 2.34) < 0.001	1.42 (1.25, 1.62) < 0.001	1.52 (1.31, 1.76) < 0.001

^a^Model I adjust for age

^b^Model II adjust for gender, age, BMI, DBP, ABSI, and smoking, if not be stratified

*LDL-C* low-density lipoprotein cholesterol, *MetS* metabolic syndrome, *BMI* body mass index, *DBP* diastolic blood pressure, *ABSI* a body shape index

Since there was a U-shaped association between the serum LDL-C levels and MetS prevalence in the whole studied population and male cohort, the thresholds were further evaluated using piecewise linear regression analysis. The prevalence of MetS increased with increasing level of the serum LDL-C above the inflection point (LDL-C concentration = 113.6 mg/dL) in the overall population (OR, 1.09; 95% CI, 1.01–1.17; *P* < 0.05). The inflection point of serum LDL-C in male cohort was detected at the concentration of 117.6 mg/dL, above which no statistically significant difference was observed between increased LDL-C level and MetS prevalence as compared to those with the LDL-C levels below the inflection point (OR, 1.05; 95% CI, 0.97–1.15; *P* > 0.05) (Table 3). In addition, as compared to that of low LDL-C levels (< 135.0 mg/dL), the OR (95% CI) of high LDL-C levels (> 135.0 mg/dL) in MetS prevalence was 1.55 (1.36–1.77) in female workers in Modell II (Table 3).

**Table 3:** Multivariable regression analysis of serum LDL-C levels and current MetS, by inflection points

Inflection points of LDL-C (mg/dL)	Odds Ratio (95% Confidence Interval) ***P***-value		
	Crude	Model I^a^	Model II^b^
**Total (n = 60,799)**			
< 113.6	Ref.	Ref.	Ref.
≥ 113.6	1.66 (1.56, 1.76) < 0.001	1.09 (1.02, 1.16) 0.010	1.09 (1.01, 1.17) 0.019
**Men (n = 34,827)**			
< 117.6	Ref.	Ref.	Ref.
≥ 117.6	1.45 (1.35, 1.55) < 0.001	1.02 (0.95, 1.10) 0.551	1.05 (0.97, 1.15) 0.212
**Women (n = 25,972)**			
< 135.0	Ref.	Ref.	Ref.
≥ 135.0	2.66 (2.38, 2.96) < 0.001	1.53 (1.36, 1.72) < 0.001	1.55 (1.36, 1.77) < 0.001

^a^Model I adjust for age

^b^Model II adjust for gender, age, BMI, DBP, ABSI, and smoking, if not be stratified

*LDL-C* low-density lipoprotein cholesterol, *MetS* metabolic syndrome, *BMI* body mass index, *DBP* diastolic blood pressure, *ABSI* a body shape index

## Discussion

This study was conducted on 60,799 individuals from a working population cohort, and showed a U-shaped association between the serum levels LDL-C and MetS prevalence, suggesting that both the low and high levels of serum LDL-C were related to increased MetS prevalence. This relationship pattern between the two was also observed among the male workers. However, in case of female cohort, only the high LDL-C levels (> 135.0 mg/dL) increased MetS prevalence. These findings might have implications in the management of serum LDL-C in subjects with MetS during clinical practice.

Several previous studies tried to reveal the association between serum LDL-C and MetS but the conclusion are controversial. A cross-sectional study by Hajian-Tilaki et al., conducted on the representative samples of Iranian adults (*n* = 1000) aged within the range of 20–70 years, reported that there was no significant correlation between the serum LDL-C levels and MetS prevalence [11]. However, in a cross-sectional (*n* = 3871) and longitudinal (*n* = 2558) health screening study conducted in Japanese people, Oda pointed out that the LDL-C levels were related to MetS and can serve as a predictor for its development [15]. In addition, in a prospective study in China, considering a sample of 4542 subjects aged within the range of 19–80 years, Wang et al. also suggested that the increased levels of LDL-C could increase the risk of MetS [12].

Lipids play an important role in the development of MetS [16, 17]. Regarding the mechanism of how the high levels of LDL-C led to an increased MetS risk, previous studies suggested that the increased levels of serum apolipoprotein-B and circulating oxidized LDL might contribute to endothelial dysfunction and vascular inflammation by increasing the LDL-C levels [18–21]. Although the underlying mechanism, regulating an increase in the prevalence of MetS by low LDL-C levels, observed in this study remains unclear, a similar U-shaped relationship between serum LDL-C concentration and all-cause mortality has been previously reported [22, 23]. In a prospective cohort study conducted on a general population in Denmark, Johannesen et al. revealed that both the low (< 70 mg/dL) and high (> 189 mg/dL) levels of serum LDL-C were related to an elevated risk of all-cause mortality when compared to the mortality due to the moderate levels of LDL-C (132–154 mg/dL) [22]. In addition, by a large sample in Korea, Sung et al. found that the low levels of LDL-C (< 70 mg/dL) were strongly associated with the increasing risk of cancer, CVD, and all-cause mortality [23]. These findings indicated that the clinicians need to pay attention to the effects of low levels of LDL-C on diseases.

The results of this study, revealing the relationship between the high levels of serum LDL-C and MetS prevalence, were in agreement with several previous studies [12, 15]. Of note, this study demonstrated that the low levels of LDL-C (< 103.8 mg/dL) could also increase MetS prevalence in the overall population. A further investigation into these results suggested that the gender might influence the association between the two. The male participants accounted for 57.3% of the total studied population and showed a significant U-shaped association between the levels of serum LDL-C and MetS prevalence, while in the female cohort, only the high levels of LDL-C (> 135.0 mg/dL) were observed to be related to an increased MetS prevalence. These findings indicated that the gender-specific management of LDL-C levels might be needed to prevent the development of MetS, such as the maintenance of lower LDL-C levels might be beneficial for women, but not necessarily for men.

### Study strength and limitations

The strengths of the present study included a large study sample, and rigorous statistical analyses. However, there were several limitations as well, which should be addressed. Firstly, the participants were recruited from a working population in a single region in Spain, thus, the results might be not be extrapolated to general population, non-Caucasian ethnicities, or other countries or regions. Secondly, the findings of this study do not indicate that the MetS diagnosed using other diagnostic criteria (e.g., the diagnostic criteria of MetS by the World Health Organization, the International Diabetes Federation, or the European Group for the Study of Insulin Resistance) would also show this pattern of relationship with LDL-C. Thirdly, since this study was just a secondary analysis based on a cross-sectional study, it could not deduce causality between the LDL-C level and current MetS statuses. In addition, the lack of several variables (such as co-existing disease, lipid lowering treatment or other drug use, and alcohol use) in the raw data limited a further subgroup analysis.

## Conclusions

Both the low and high levels of serum LDL-C were observed to be related to the increasing prevalence of MetS both in the working population and male workers. However, only the high LDL-C levels were shown to be positively associated with an increase in the prevalence of MetS in female workers. These findings revealed that the management of LDL-C should be carried out taking into consideration the gender-specific differences in MetS. These findings also indicated that, in order to reduce the prevalence of MetS, it might be appropriate to pursue the lower levels of LDL-C among female population rather than male population.

## Data Availability

The data used in this study can be downloaded from ‘DATADRYAD’ database (www.Datadryad.org).

## Abbreviations

MetS: Metabolic syndrome
CVD: Cardiovascular disease
HDL-C: High-density lipoprotein cholesterol
LDL-C: Low-density lipoprotein cholesterol
BMI: Body mass index
WHtR: Waist-to-height ratio
WC: Waist circumference
BF: Body fat
ABSI: A body shape index
SBP: Systolic blood pressure
DBP: Diastolic blood pressure
TC: Total cholesterol
FBG: Fasting blood glucose

## References

[1] Hirode G, Wong RJ (2020). Trends in the prevalence of metabolic syndrome in the United States, 2011-2016. JAMA..

[2] Bishehsari F, Voigt RM, Keshavarzian A (2020). Circadian rhythms and the gut microbiota: from the metabolic syndrome to cancer. Nat Rev Endocrinol.

[3] Rochlani Y, Pothineni NV, Kovelamudi S, Mehta JL (2017). Metabolic syndrome: pathophysiology, management, and modulation by natural compounds. Ther Adv Cardiovasc Dis.

[4] Castro-Barquero S, Ruiz-León AM, Sierra-Pérez M, Estruch R, Casas R (2020). Dietary strategies for metabolic syndrome: a comprehensive review. Nutrients..

[5] Beltran-Sanchez H, Harhay MO, Harhay MM, McElligott S (2013). Prevalence and trends of metabolic syndrome in the adult US population, 1999-2010. J Am Coll Cardiol.

[6] Gu D, Reynolds K, Wu X, Chen J, Duan X, Reynolds RF, Whelton PK, He J, InterASIA collaborative group (2005). Prevalence of the metabolic syndrome and overweight among adults in China. Lancet..

[7] Dregan A, Rayner L, Davis KAS, Bakolis I, de la Torre JA, Das-Munshi J, Hatch SL, Stewart R, Hotopf M (2020). Associations between depression, arterial stiffness, and metabolic syndrome among adults in the UK biobank population study: a mediation analysis. JAMA Psychiatry.

[8] Gill MG, Majumdar A (2020). Metabolic associated fatty liver disease: addressing a new era in liver transplantation. World J Hepatol.

[9] Wang S, Tu J, Pan Y (2019). Threshold effects in the relationship between serum non-high-density lipoprotein cholesterol and metabolic syndrome. Diabetes Metab Syndr Obes.

[10] Sampson M, Ling C, Sun Q, Harb R, Ashmaig M, Warnick R, Sethi A, Fleming JK, Otvos JD, Meeusen JW, Delaney SR, Jaffe AS, Shamburek R, Amar M, Remaley AT (2020). A new equation for calculation of low-density lipoprotein cholesterol in patients with normolipidemia and/or hypertriglyceridemia. JAMA Cardiol.

[11] Hajian-Tilaki K, Heidari B, Hajian-Tilaki A, Firouzjahi A, Bakhtiari A (2017). Does the low-density lipoprotein cholesterol play a key role in predicting metabolic syndrome in the Iranian adult population?. Caspian J Intern Med.

[12] Wang XR, Song GR, Li M, Sun HG, Fan YJ, Liu Y, Liu QG (2018). Longitudinal associations of high-density lipoprotein cholesterol or low-density lipoprotein cholesterol with metabolic syndrome in the Chinese population: a prospective cohort study. BMJ Open.

[13] Romero-Saldaña M, Tauler P, Vaquero-Abellán M, López-González AA, Fuentes-Jiménez FJ, Aguiló A, Álvarez-Fernández C, Molina-Recio G, Bennasar-Veny M (2018). Validation of a non-invasive method for the early detection of metabolic syndrome: a diagnostic accuracy test in a working population. BMJ Open.

[14] Wang S, Zhang J, Lu X (2019). Non-linear association of plasma level of high-density lipoprotein cholesterol with endobronchial biopsy bleeding in patients with lung cancer. Lipids Health Dis.

[15] Oda E (2013). Low-density lipoprotein cholesterol is a predictor of metabolic syndrome in a Japanese health screening population. Intern Med.

[16] Monnerie S, Comte B, Ziegler D, Morais JA, Pujos-Guillot E, Gaudreau P (2020). Metabolomic and lipidomic signatures of metabolic syndrome and its physiological components in adults: a systematic review. Sci Rep.

[17] Surowiec I, Noordam R, Bennett K, Beekman M, Slagboom PE, Lundstedt T, van Heemst D (2019). Metabolomic and lipidomic assessment of the metabolic syndrome in Dutch middle-aged individuals reveals novel biological signatures separating health and disease. Metabolomics..

[18] Williams K, Sniderman AD, Sattar N, D'Agostino R, Wagenknecht LE, Haffner SM (2003). Comparison of the associations of apolipoprotein B and low-density lipoprotein cholesterol with other cardiovascular risk factors in the insulin resistance atherosclerosis study (IRAS). Circulation..

[19] Holvoet P, Lee DH, Steffes M, Gross M, Jacobs DR (2008). Association between circulating oxidized low-density lipoprotein and incidence of the metabolic syndrome. JAMA..

[20] Gragnano F, Calabrò P (2018). Role of dual lipid-lowering therapy in coronary atherosclerosis regression: evidence from recent studies. Atherosclerosis..

[21] Gragnano F, Fimiani F, Di Maio M, Cesaro A, Limongelli G, Cattano D, Calabrò P (2019). Impact of lipoprotein(a) levels on recurrent cardiovascular events in patients with premature coronary artery disease. Intern Emerg Med.

[22] Johannesen CDL, Langsted A, Mortensen MB, Nordestgaard BG (2020). Association between low density lipoprotein and all cause and cause specific mortality in Denmark: prospective cohort study. BMJ..

[23] Sung KC, Huh JH, Ryu S, Lee JY, Scorletti E, Byrne CD, Kim JY, Hyun DS, Ko SB (2019). Low levels of low-density lipoprotein cholesterol and mortality outcomes in non-statin users. J Clin Med.

